# Dietary Intervention Accelerates NASH Resolution Depending on Inflammatory Status with Minor Additive Effects on Hepatic Injury by Vitamin E Supplementation

**DOI:** 10.3390/antiox9090808

**Published:** 2020-09-01

**Authors:** Julie Hviid Klaebel, Günaj Rakipovski, Birgitte Andersen, Jens Lykkesfeldt, Pernille Tveden-Nyborg

**Affiliations:** 1 Department of Veterinary and Animal Sciences, Faculty of Health and Medical Sciences, University of Copenhagen, Ridebanevej 9, 1870 Frederiksberg C, Denmark; juliehviid@sund.ku.dk (J.H.K.); jopl@sund.ku.dk (J.L.); 2 CV Research, Global Research, Novo Nordisk A/S, Novo Nordisk Park 1, 2670 Måløv, Denmark; gura@novonordisk.com; 3 Liver Disease Research, Global Research, Novo Nordisk A/S, Novo Nordisk Park 1, 2670 Måløv, Denmark; btta@novonordisk.com

**Keywords:** fibrosis, hepatic hallmarks, lifestyle modifications, nonalcoholic fatty liver disease, nonalcoholic steatohepatitis, vitamin E

## Abstract

Despite the lack of effective pharmacotherapy against nonalcoholic steatohepatitis (NASH) and liver fibrosis, vitamin E (vitE) supplementation and lifestyle modifications are recommended for the management of NASH due to promising clinical results. We recently reported a positive effect of supplementation with 800 IU vitE and atorvastatin on NASH resolution in guinea pigs. In the present study, we investigated the effect of high-dose vitE therapy combined with dietary intervention against progressive NASH and advanced fibrosis in the guinea pig model. Sixty-six guinea pigs received either high-fat (HF) or standard guinea pig chow diet (Control) for 25 weeks. Prior to eight weeks of intervention, HF animals were allocated into groups; dietary intervention (Chow) or dietary intervention with 2000 IU/d vitE supplementation (CvitE). Both Chow and CvitE reduced dyslipidemia, hepatic lipid accumulation and liver weight (*p* < 0.05), while CvitE further decreased hepatocellular ballooning (*p* < 0.05). Subanalyses of individual responses within intervention groups showed significant correlation between the hepatic hallmarks of NASH and lipid accumulation vs. inflammatory state (*p* < 0.05). Collectively, our results indicate that individual differences in sensitivity towards intervention and inflammatory status determine the potential beneficial effect of dietary intervention and high-dose vitE supplementation. Moreover, the study suggests that inflammation is a primary target in NASH treatment.

## 1. Introduction

Nonalcoholic steatohepatitis (NASH) constitutes the progressed form of nonalcoholic fatty liver disease (NAFLD) [1]. NASH is estimated to affect 1.5–6.5% of the general population, and is presently a leading cause of liver transplantations in the United States [2,3]. Several factors are part of the etiology of this complex disease and the progression from NAFLD to NASH. Of these, a chronic state of dyslipidemia, sustained by a diet high in fat and sugars, constitutes an important contributor in most patients, promoting hepatic steatosis and the accumulation of hepatic lipotoxic lipids, leading to oxidative stress, inflammation and steatohepatitis denoting NASH [4,5,6]. The causative hepatocellular changes induce an overproduction of reactive oxygen species, thereby exceeding the antioxidant capacity to quench free radicals and generating oxidative stress [6,7]. The result is damage to cellular organelles and membranes, and the induction of inflammation by promoting the release of inflammatory cytokines, including transforming growth factor-β (TGF-β), tumor necrosis factor-α (TNF-α) and interleukin (IL)-8 [8]. Increased levels of reactive oxygen species induce the activation of TGF-β and subsequent activation of hepatic stellate cells governing the deposition of a fibrotic extracellular matrix consequently leading to hepatic fibrosis [9,10], which may be slowed by antioxidants [11,12]. As the disease progresses, the initial and relatively simple state of steatosis advances to hepatitis (NASH) with increasing cellular damage and apoptosis, promoting liver fibrosis and potentially life-threatening liver failure [2,3,7,13]. In NASH patients, the oxidant/antioxidant equilibrium has previously been found to be impaired, with disease severity correlated to decreased antioxidants and increased reactive oxygen species [14,15]. As a potent antioxidant, vitamin E’s (vitE) mechanism of action in NASH resolution is proposedly through the reduction of oxidative stress and accompanying cell damage [11].

As disease prevalence is rapidly increasing, so is the need for finding effective pharmacological treatment modalities [4,16]. First line management in patients constitutes life style changes, where dietary restrictions and weight loss has been shown to induce positive effects on hepatic hallmarks of NASH in both patients and animal models [17,18,19,20]. However, to achieve an improvement of a minimum of “1” in hepatic inflammatory and fibrosis score, sustained weight loss above 10% has been shown to be necessary [21,22]. Unfortunately, life style therapy is rarely satisfactory, reports showing that less than 10% of patients are able to successfully accomplish and sustain a weight loss of 10% or above, emphasizing the need for pharmacological therapy combined with life style modifications [23]. Currently, vitE therapy of 800 IU/day is recommended in patients diagnosed with NASH, including patients with hepatic fibrosis and/or increased risk of fibrosis progression, not suffering from diabetes and cirrhosis [2,24]. In both adults and children (PIVENS and TONIC trials, respectively) vitE supplementation is reported to decrease NAFLD histopathology, including resolution of inflammatory hallmarks, compared to non-supplemented controls [25,26,27]. This is supported by meta-analyses of clinical trials, where vitE has been shown to decrease aminotransferase levels (hepatic injury enzymes), improve steatosis, lobular inflammation, ballooning hepatocytes, and to be effective in the overall resolution of steatohepatitis [28,29]. Additional mechanisms of vitE supplementation have been proposed, including regulation of the inflammatory response, cell proliferation and regulation of the expression of target genes coupled to the formation of hepatic damage in NASH [30,31]. VitE putatively possesses the ability to inhibit the activity of protein kinase C, resulting in reduced proliferation and adhesion of various cell types, including monocytes/macrophages and neutrophils hallmarking NASH, as well as fibroblasts involved in the deposition of extracellular fibrosis [11,30,31]. Moreover, vitE has been suggested to affect key cellular pathways in NASH by regulating the expression of target genes involved in the formation of steatosis (HMG-CoA reductase, a low-density lipoprotein receptor and scavenger receptor cluster of differentiation 36), inflammation (I-2, IL-4, IL-1β and nuclear factor-κΒ) and fibrosis (TGF-β, collagen 1a1 (Col1a1) and matrix metalloproteinases) [20,30,32,33]. On the other hand, results on the effects of vitE therapy on hepatic fibrosis regression has not been conclusive. A positive trend towards fibrosis regression was observed in the preclinical guinea pig model subjected to vitE (800 IU/d) and atorvastatin (1 mg/kg) treatment, mimicking the doses applied in humans [20,28,34]. In this study, vitE and atorvastatin treatment combined with dietary intervention resulted in an increased effect on ballooning hepatocytes, lobular inflammation and fibrosis compared with dietary intervention alone [20]. *q*PCR expression analyses showed a significant decrease in inflammatory (IL-8 and monocyte chemotactic protein 1 (MCP-1)) and fibrotic target genes (TGF-β, α smooth muscle actin (α-sma) and Col1a1) in NASH animals subjected to vitE supplementation and diet change (low fat, no cholesterol), compared to a dietary intervention alone [20]. Findings supported dietary intervention to be a prerequisite for the positive effects presumably induced by vitE and atorvastatin treatment [20]. However, the study did not investigate the isolated effects of vitE monotherapy.

With offset in previous findings, the present study explores the isolated effects of an increased vitE dose on NASH hallmarks (hepatic injury, inflammation and fibrosis) compared with a change in diet. In humans, the reports of potential risks of vitE supplementation are conflicting; an increase in all-cause mortality has been reported with vitE doses above 400 IU/d whereas a meta-analysis from Abner et al. found no association between vitE doses below 5500 IU/d and mortality rate [35,36]. Applying the validated guinea pig NASH model, the beneficial role of a diet change from a NASH-inducing high fat/high cholesterol diet to a low fat/no cholesterol diet, with and without a high-dose (2000 IU/d) vitE supplementation in a progressive disease state is evaluated. Endpoints include effects on histopathological hallmarks of NASH and biochemical markers of dyslipidemia and vitE status, and molecular markers of target genes associated with inflammation, fibrosis and hepatic lipid metabolism [37,38].

## 2. Materials and Methods

### 2.1. Animals and Experimental Design

All animal experimentation was approved by the Animal Experimentation Inspectorate under the Danish Ministry of Environment and Food, and in accordance with the European Legislation of Animal Experimentation 2010/63/EU.

**Figure 1:**
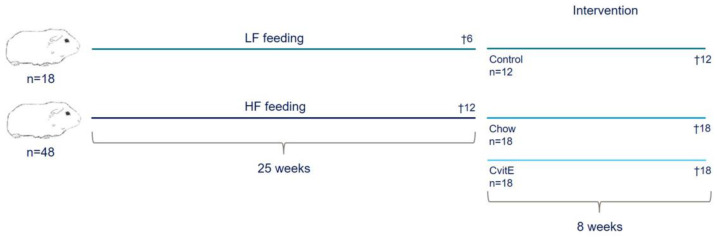
Study overview. Sixty-six guinea pigs were preloaded on either a standard guinea pig chow diet (4% fat, 0% cholesterol, and 0% sucrose) or high-fat diet (20% fat, 0.35% cholesterol, and 15% sucrose) for 25 weeks. Six animals receiving standard guinea pig chow diet and 12 animals receiving high-fat diet were randomly selected for euthanasia prior to intervention start, providing baseline values. The remaining animals were weight stratified into: Control (*n* = 12), proceeding on the standard guinea pig chow diet (4% fat, 0% cholesterol, and 0% sucrose); Chow (*n* = 18), receiving the standard guinea pig chow diet and CvitE (*n* = 18), receiving the standard guinea pig chow diet supplemented with vitamin E (1125 mg/kg feed all-rac vitamin E corresponding to a daily intake of 2000 IU vitE per animal). After eight weeks of intervention, guinea pigs were euthanized. HF, high-fat diet; LF, low-fat diet.

Sixty-six female Dunkin Hartley guinea pigs (Envigo, Indianapolis, IN, USA), weighing 400–500 g, were included in the study. At arrival, all animals were tagged with a subcutaneous microchip (E-Vet, Haderslev, Denmark) for identification. Animals were housed in groups in large floor pens, with wood shavings as bedding material and access to hay and environmental enrichment. Feed and water was offered ad libitum. Animals were kept on a 12 h light−dark cycle and at a room temperature between 20–24 °C. Animal caretakers inspected all animals daily, and body weights were monitored once a week throughout the study period. Prior to engaging in the study, animals were allowed one week of acclimatization on a standard guinea pig chow diet. At study start, the guinea pigs were randomized to two weight stratified groups, receiving either a standard guinea pig chow diet (control; 4% fat, 0% cholesterol, 0% sucrose; 18 animals) or a high-fat diet (HF; 20% fat, 0.35% cholesterol, 15% sucrose; 48 animals) for 25 weeks. Six control animals and 12 HF animals were randomly selected for euthanasia prior to intervention start at week 25, providing preintervention baseline values (Control25 and HF, respectively). The remaining control animals (*n* = 12) continued in their group, while HF animals were weight stratified into two groups (*n* = 18): one group switched to the standard guinea pig chow diet (Chow; equivalent to the control diet) and the other switched to a standard guinea pig chow diet supplemented with 1125 mg/kg feed all-rac vitE corresponding to a daily intake of 2000 IU vitE (CvitE) per animal. Study overview is provided in Figure 1. Diets were manufactured by Ssniff (Ssniff Spezialdiäten, Soest, Germany) and stored at −20 °C. To limit auto-oxidation, feed was thawed in smaller portions twice a week before feeding to the animals. Table 1 displays the dietary composition of the included diets.

After 8 weeks of intervention, animals were euthanized, according to previously performed studies [39,40]. A study overview is provided in Figure 1. In short, animals were semi-fasted overnight (i.e., only access to hay and water) before preanesthesia with 1.25 mL/kg Zoletil-mix (zoletil-50 supplemented with xylazine and butophanol) before being placed on inhalation anesthesia (5% Isoflurane, Isoba vet 100%, Intervet International). When interdigital reflexes disappeared, the animal was placed in dorsal recumbency and an intracardial blood sample collected in a 10 mL syringe flushed with 5% EDTA and in NaF and heparin coated microvettes. The animal was euthanized by decapitation immediately after blood samples were obtained.

### 2.2. Plasma Samples

For free fatty acids (FFA) and alkaline phosphatase (ALP), intracardial blood samples were collected in NaF and heparin coated microvettes (Sarstedt, Nümbrecht, Germany). For the remaining analyses, EDTA stabilized samples were used.

Immediately after collection, plasma was isolated by centrifugation at 2000× *g* for 4 min at 4 °C and stored at −20 °C prior to analyses. Total cholesterol (TC), triglyceride (TG), aspartate aminotransferase (AST), alanine aminotransferase (ALT), FFA and ALP were performed on Cobas 6000 (Roche Diagnostic Systems, Berne, Switzerland) according to manufacturer’s specifications. Plasma samples for measurement of alpha tocopherol (αToc (vitE)) were stored at −80 °C and later analyzed by HPLC as previously described [41].

### 2.3. Liver Samples

Immediately after euthanasia, the liver was isolated and quickly placed in ice cold PBS, before gently dried on paper cloth, weighed and photographed. A total of seven 0.30–5 cm thick slices spanning the left lateral lobe (lobus hepatis sinister lateralis) were obtained, with four sections designated to biochemical analyses, one section to molecular biological analyses and two sections to histological analyses.

Hepatic tissue samples were frozen in liquid nitrogen and stored at −80 °C for biochemical analyses of cholesterol (HC) and triglyceride (HT). Prior to analyses, hepatic tissue was homogenized and measured on a Cobas 6000 according to the manufacturer’s specifications, as described previously [39]. In short, 30–40 mg tissue was homogenized with 1.0 mL extraction buffer (0.15 M sodium acetate and 0.75% Triton-X) on a TissueLyser (Qiagen, Hilden, Germany) with steel-beads. Afterwards, the homogenized tissue samples were heated in a heating block at 100 °C for 2 min followed by cooling on ice and supplemented with 0.5 mL extraction buffer. Samples were then centrifuged at 9000× *g* for 10 min at 4 °C and the supernatant isolated and stored at −20 °C prior to analyses of HC and HT. Hepatic tissue for analysis of αToc content was stabilized with butylated hydroxytoluene and measured by HPLC, according to [42].

### 2.4. Histology

Liver sections were placed in plastic grid tissue containers and fixed in 10% formalin before processing and embedding in paraffin. Embedded tissue was subsequently sectioned in 2–4 µm thick slices. For histopathological examination of steatosis, lobular inflammation and ballooning hepatocytes sections were stained with haematoxylin and eosin and picro sirius red for evaluation of fibrosis [20]. Liver slides were scored in a blinded fashion using a semiquantitative scoring system based on the methodology developed by Kleiner et al. [43]. Steatosis was assessed in the entire liver section and graded: 0 (<5%), 1 (5–33%), 2 (>33–66%) or 3 (>66%). The presence of lobular inflammation and ballooning hepatocytes was assessed in 20 randomly selected fields of view, at a ×20 zoom, using the Visiopharm software (Visiopharm, version 2018.9.4.5608, Visiopharm A/S, Hørsholm, Denmark). Lobular inflammation was graded using the average number of recorded foci pr. field; 0 (not present), 1 (<2 foci), 2 (2–4 foci) or 3 (>4 foci). A focus was defined as a cluster of 3 or more inflammatory cells. Ballooning hepatocytes were graded as 0 (not present), 1 (few) or 2 (many/prominent). The distribution of hepatic fibrosis was assessed in the entire liver section and graded as: 0 (not present), 1 (mild, zone 3, perisinusoidal; moderate, zone 3, perisinusoidal; portal/periportal), 2 (perisinusoidal and portal/periportal), 3 (bridging) and 4 (cirrhosis). Portal inflammation was graded as 0 (not present) or 1 (present), with presence defined as ≥2 portal areas containing ≥2 foci, with foci defined as ≥5 inflammatory cells. Finally, the NAFLD activity score (NAS) was calculated consisting of the sum of steatosis grade, lobular inflammation grade and ballooning hepatocyte grade, with a range between a minimum of 0 and a maximum score of 8 [20,43].

### 2.5. qPCR

Liver samples designated for *q*PCR analyses were flash frozen in liquid nitrogen and stored at −80 °C until use. Execution of RNA extraction, reverse transcription and *q*PCR analyses were performed as previously described [37]. Briefly, 1000 µL MagMax Lysis/Binding Solution Concentrate (Thermo Fisher, Waltham, MA, USA) and 0.7% β-mercaptoethanol (Sigma Aldrich, St. Louis, MO, USA) were used to homogenize 50 mg liver tissue. The homogenate was centrifuged for 1 min at 10.000 RPM at 4 °C followed by isolation of the supernatant, which was afterwards stored in RNAse-free Eppendorf tubes and frozen for 24 h at −20 °C. Purification of RNA was done with the MagMax-96 Total RNA Isolation Kit (Thermo Fisher, Waltham, MA, USA) according to manufacturer’s specifications. To synthesize cDNA, 500 ng RNA was subjected to reverse transcription (High Capacity cDNA Reverse Transcription Kit; Thermo Fisher, Waltham, MA, USA) on a 2720 Thermal Cycler (Applied Biosystems, Foster City, CA, USA) at 25 °C for 10 min, 37 °C for 120 min, 85 °C 5 s. Prior to further analyses cDNA was controlled for genomic DNA contamination using an intron-spanning primer set (β-actin) [44].

*q*PCR analyses were performed by mixing 2 µL undiluted cDNA with 8 µL Master Mix (5 µL PowerUp SYBR Green Master Mix (Thermo Fisher, Waltham, MA, USA), 1 µL primer mix and 2 µL RNAse-free water). All samples were run in triplicates applying the StepOnePlus Real Time PCR system (Applied Biosystems, Foster City, CA, USA) at 50 °C for 2 min, 95 °C for 5 min and then in 40 cycles (95 °C for 10 s, 60 °C for 10 s and 72 °C for 20 s) before cooling to 4 °C. For tumor necrosis factor α (TNF-α) and IL-8 the annealing step of the *q*PCR cycle was executed at 62 °C and not 60 °C. Hypoxanthine phosphoribosyltransferase was used as reference gene [20,37]. Primer details are listed in Table 2.

### 2.6. Statistical Analyses

Statistical analyses were performed in GraphPad Prism 8 (GraphPad Software, La Jolla, CA, USA).

Markers of plasma and hepatic lipids, liver injury enzymes, liver weight, αToc and expression of target genes were analyzed by one-way ANOVA with Sidak’s post hoc test. Data are presented as means with standard deviations (SD). If inhomogeneous variances were observed, data sets were log-transformed before analysis and then back-transformed and presented as geometric means with 95% confidence intervals. Data analyses of liver histology were done by Kruskal−Wallis nonparametric test with Dunn’s post hoc test, presented as individual values with medians. Histological differences between the preintervention groups (HF and control25) were analyzed by the nonparametric Mann−Whitney U test.

Subanalyses of Chow and CvitE groups were performed by subdividing each group according to their inflammation score: low (score 0), intermediate (score 1) and high (score 2–3). Subanalyses of TNF-α, IL-8, MCP-1, α-sma, Col1a1, TGF-β, cytochrome P450 7A1 (CYP7A1), HC and HT were analyzed by ordinary one-way ANOVA with Sidak’s post hoc test. Subanalyses of steatosis, ballooning hepatocytes, fibrosis and NAS were done by Kruskal−Wallis nonparametric test with Dunn’s post hoc test. Furthermore, correlation analyses between inflammation and steatosis, ballooning hepatocytes, fibrosis, NAS, TNF-α, IL-8, MCP-1, α-sma, Col1a1, TGF-β, CYP7A1, HC and HT were calculated using the nonparametric Spearman’s correlation. A *p*-value below 0.05 was considered statistically significant.

## 3. Results

### 3.1. Dyslipidemia

After 25 weeks of high-fat feeding, the animals proved dyslipidemic with significantly increased levels of TC (*p* < 0.0001) compared with Control25 animals euthanized prior to the start of intervention. Moreover, Control25 showed significantly increased TG, FFA and ALP levels compared with HF (*p* < 0.0001, *p* < 0.05, *p* = 0.05). Dietary intervention and vitE treatment resulted in reduced TC levels in Chow and CvitE compared with HF after 8 weeks of intervention, although only Chow reached significance (*p* < 0.05). Both Chow and CvitE showed significantly elevated TC levels compared with Control (*p* < 0.0001 and *p* < 0.0001). TG levels of the Control group were significantly increased compared with HF, Chow and CvitE (*p* < 0.0001). During the intervention period, no differences were observed in FFA and ALP levels between groups. See Table 3 for overview of plasma results.

### 3.2. Plasma Biochemical Markers

Control25 showed decreased levels of the hepatic injury enzymes ALT and AST compared with the HF group after 25 weeks of high-fat feeding, although only AST reached significance (*p* < 0.001). After 8 weeks of intervention, no improvement in ALT levels were detected in Chow and CvitE compared to HF, while the two groups were significantly increased compared with Control (*p* < 0.05 and *p* < 0.05). However, an improvement in AST level was detected in chow and CvitE, both being significantly reduced compared with HF (*p* < 0.01 and *p* < 0.05). Compared with Control, the two intervention groups showed elevated AST levels, although only CvitE was significantly increased (*p* < 0.01). αToc levels were highly increased in CvitE animals compared with HF, Control and Chow (*p* < 0.0001, for all groups), reflecting the dietary vitE supplement. See Table 3 for overview of plasma results.

### 3.3. Liver Status

Accumulation of lipids in the liver was evident after the high-fat induction period, where the relative liver weight, HC and HT was significantly increased in HF animals compared with Control25 (*p* < 0.0001, for all three parameters). The liver weight of both chow and CvitE was significantly reduced after 8 weeks of intervention compared with the HF group (*p* < 0.0001), although different from the control group (*p* < 0.0001). Likewise, Chow and CvitE showed significant reduction in HC and HT compared with HF (chow: *p* < 0.001, *p* < 0.0001; CvitE: *p* < 0.001, *p* < 0.0001), but were significantly increased compared with Control (chow: *p* = 0.01, *p* < 0.0001; CvitE: *p* < 0.01, *p* < 0.0001).

Hepatic αToc levels reflected plasma concentrations; CvitE group showing an increase compared with HF, Control and Chow (*p* < 0.0001), supporting an effective absorption of vitE in hepatic tissue. An increased αToc level was found in the Chow group compared with Control (*p* < 0.01). See Table 4 for overview.

### 3.4. Histopathological Evaluation

By week 25, the HF diet successfully induced hepatic lesions characteristic of NASH, with steatosis (*p* < 0.001), lobular inflammation (*p* < 0.01), ballooning hepatocytes (*p* < 0.001), NAS (*p* < 0.001) and fibrosis (*p* < 0.01) compared with Control25. Furthermore, the high-fat feeding regimen also resulted in advanced bridging fibrosis in the HF group (five animals displayed F3 fibrosis stage at baseline).

**Figure 2:**
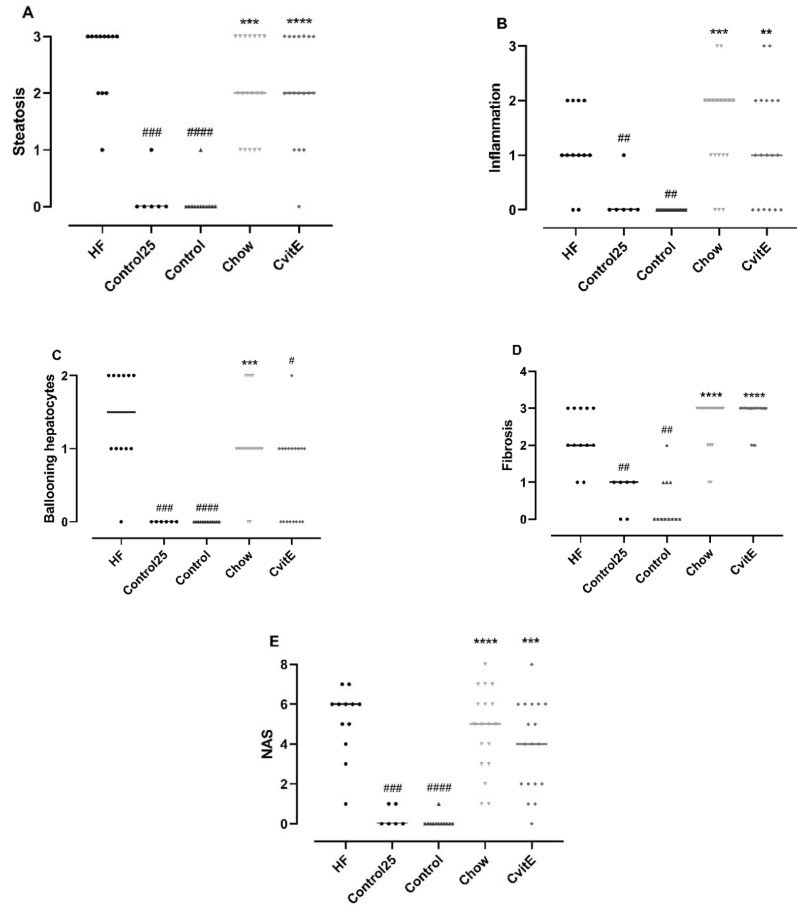
Histopathological evaluation of disease severity. (**A**) Steatosis: Control25 and Control animals showed significantly lower steatosis grade compared with HF. Chow and CvitE showed significantly increased steatosis compared with Control and were not different from HF. (**B**) Inflammation: Control25 and Control were significantly decreased compared with HF, while Chow and CvitE were significantly increased compared with Control with no difference compared to HF. (**C**) Ballooning hepatocytes; Control25 and Control were significantly different from HF and Chow significantly different from Control. CvitE was significantly reduced compared with HF and not different from Control. (**D**) Fibrosis; Control25 and Control had a significantly lower fibrosis score compared with HF and Chow and CvitE had a significantly increased fibrosis score compared with Control with no difference compared to HF. (**E**) NAFLD activity score (NAS): in the evaluation of disease severity no effect of treatment was observed in Chow and CvitE, which was not different from HF and significantly increased compared with Control. Control25 and Control were significantly reduced compared with HF. Data are presented as individual values with medians. ** *p* < 0.01, *** *p* < 0.001, **** *p* < 0.0001, compared with Control; ^#^ *p* < 0.05, ^##^ *p* < 0.01, ^###^ *p* < 0.001, ^####^ *p* < 0.0001, compared with HF.

After 8 weeks of intervention, ballooning cells were significantly reduced in CvitE animals compared with HF animals (*p* < 0.05). For the other histopathological scoring parameters, the groups (Chow, CvitE) were not significantly different from the HF group, but—as expected—all groups differed significantly from controls (*p* < 0.01). See Figure 2.

### 3.5. Expression of Target Genes/qPCR

**Figure 3:**
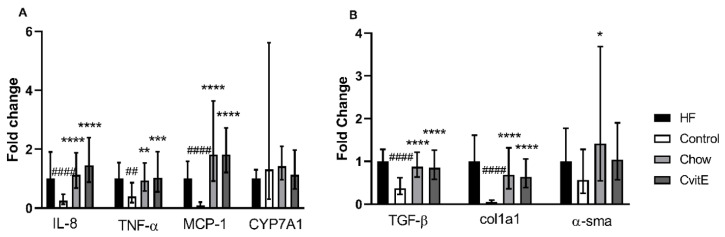
Expression of key target genes. (**A**) Expression of inflammatory target genes (IL-8, TNF-α and MCP-1) showed a significant downregulation in the Control group compared with HF. Furthermore, expression levels in Chow and CvitE were significantly elevated compared with Control, while no difference compared to HF was detected. CYP7A1 expression levels did not show any difference between groups. (**B**) Expression of the fibrotic target genes TGF-β and Col1a1 showed significant downregulation in the Control group compared with HF, while chow and CvitE showed significant upregulation compared with Control with no difference to HF. In the expression of α-sma, Chow was significantly upregulated compared with Control. Moreover, the Control group appeared downregulated compared with HF and CvitE although not reaching significance. * *p* < 0.05, ** *p* < 0.01, *** *p* < 0.001, **** *p* < 0.0001, compared with Control; ^##^ *p* < 0.01, ^####^ *p* < 0.0001, compared with HF. IL-8, interleukin 8; TNF-α, tumor necrosis factor α; MCP-1, monocyte chemotactic protein 1; CYP7A1, cytochrome P450 7A1; TGF-β, transforming growth factor β; Col1a1, collagen 1a1; α-sma, α smooth muscle actin.

To evaluate inflammatory markers, the expression of IL-8, TNF-α and MCP-1 was assessed. IL-8 expression in the Control group was downregulated by 0.75 fold compared with HF (*p* < 0.0001), and the Chow and CvitE group upregulated with 0.85 and 1.15 fold, respectively, compared with Control (*p* < 0.0001, for both groups), with no difference detected between the intervention groups and the HF group. TNF-α expression was not different in HF, Chow and CvitE, but increased by 0.6 fold compared with Control (chow *p* < 0.01, CvitE *p* < 0.001 and HF *p* < 0.01). Expression of MCP-1 showed no difference between HF, Chow and CvitE, while being upregulated up to 1.7 fold compared with Control (*p* < 0.0001, for all three groups). See Figure 3.

Profibrotic growth factors, hepatic stellate cell activation and extracellular matrix deposition were assessed by the expression of TGF-β, α-sma and Col1a1, respectively. No difference was detected in the expression of TGF-β between Chow, CvitE and HF, while Control was significantly downregulated by 0.5-, 0.5- and 0.7-fold, respectively, (*p* < 0.0001, for all groups). This also applied to the expression of Col1a1, with Control being significantly downregulated compared with Chow, CvitE and HF by 0.5-, 0.5- and 0.9-fold, respectively, (*p* < 0.0001, for all groups) and no difference recorded between the intervention groups and HF. In the expression of α-sma, although the Control group appeared downregulated by 0.5-fold, compared to HF, the difference was not significant. Chow was significantly upregulated by 1.0-fold, compared with Control (*p* < 0.05), while CvitE appeared upregulated by 0.5-fold, compared to Control, although not reaching significance. Chow and CvitE α-sma expression was not different from the HF animals. See Figure 3.

The expression of the CYP7A1 gene was assessed as a marker of changes in bile acid regulation reflecting hepatic cholesterol metabolism. No difference to Control group in CYP7A1 expression could be detected, albeit may be masked by a large variation within this group. See Figure 3.

### 3.6. Subanalyses of Chow and CvitE/Correlation

To explore the relationship between hepatic inflammatory status and the effect of diet and dietary intervention combined with vitE, subanalyses of the two intervention groups (Chow and CvitE) were performed. Animals were categorized according to their individual inflammatory status; low (inflammation score 0), intermediate (inflammation score 1) and high (inflammation score 2–3). Analyses were conducted on the following outcomes: steatosis, ballooning hepatocytes, NAS-index, fibrosis, IL-8, MCP-1, TNF-α, α-sma, Col1a1, TGF-β, CYP7A1, HC and HT.

**Figure 4:**
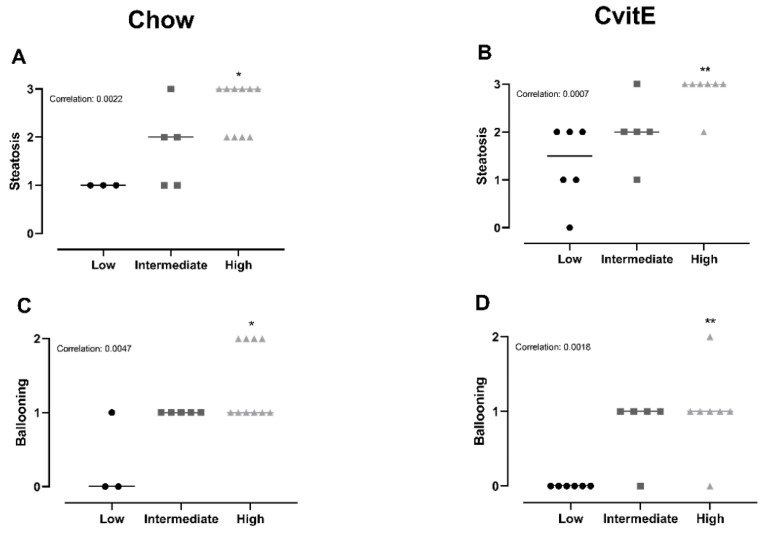

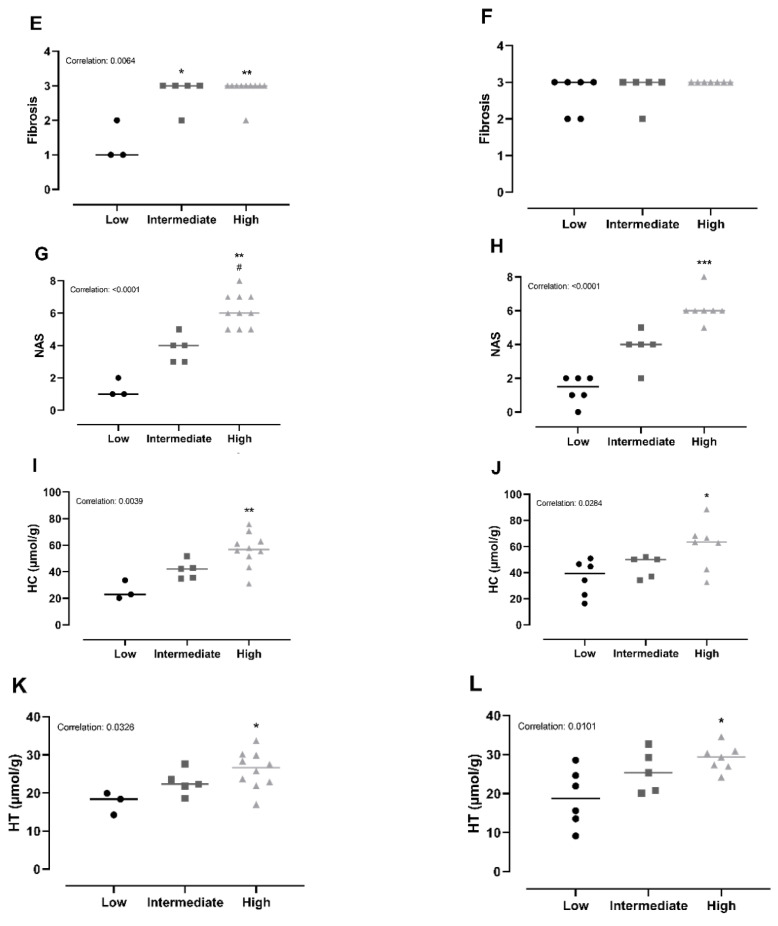
Subanalyses of histopathological markers of NASH and lipid accumulation relative to inflammatory status. (**A**,**B**) For Chow and CvitE, correlation between severity of steatosis and inflammation was detected. Low showed significantly reduced score compared with high in Chow and CvitE. (**C**,**D**) A low ballooning hepatocyte score correlated with a low inflammation score, with a significant decrease in low compared with high in Chow and CvitE. (**E**,**F**) In Chow, correlation between fibrosis and inflammation, with significant reduction in low compared with intermediate and high was detected. No correlation was observed in CvitE. (**G**,**H**) Chow and CvitE showed significant correlation between inflammation and NAS. In Chow, high was significantly increased compared with intermediate and low. In CvitE, high was significantly increased compared with low. (**I**,**J**) Correlation was observed between level of hepatic cholesterol and inflammation score in Chow and CvitE. In Chow and CvitE, low had significantly lower cholesterol levels compared with high. (**K**,**L**) Chow and CvitE showed correlation between hepatic triglyceride level and inflammation score, with significantly decreased levels in low compared with high. Data are presented as individual values with medians. * *p* < 0.05, ** *p* < 0.01, *** *p* < 0.001, compared with low; ^#^ *p* < 0.05, compared with intermediate. NAS, NAFLD activity score; HC, hepatic cholesterol; HT, hepatic triglyceride.

**Figure 5:**
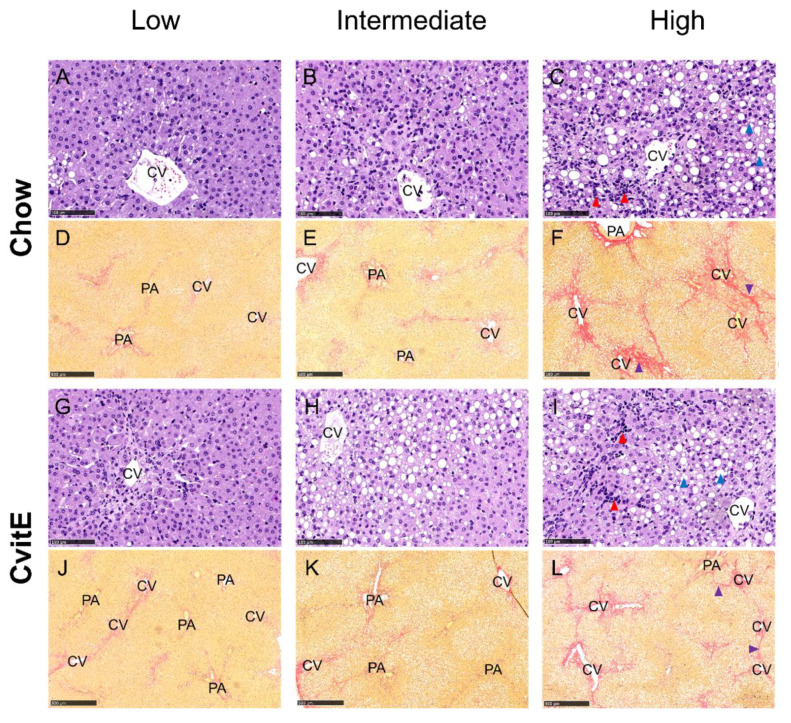
Representative histological images of liver sections at study termination. Panels are divided into subgroups: low (inflammatory score 0); intermediate (inflammatory score 1) and high (inflammatory score 2–3). (**A**–**C**,**G**–**I**) Hematoxylin and eosin stain, scale bar 100 µm. (**D**–**F**,**J**–**L**) Picro Sirius red stain (fibrosis stained red), scale bar 500 µm. The histological images show the pathological lesions associated with NASH: steatosis (macro and micro vesicular) (blue arrowheads), lobular inflammation (red arrowheads) and bridging fibrosis (purple arrowhead). Ballooning hepatocytes were also assessed, however cannot be identified at this magnification. CV, central vein; PA, portal area.

Chow animals with low inflammation scores also displayed significantly lower scores in ballooning, steatosis, NAS-index (not surprisingly) and fibrosis compared with high-inflammation counterparts (*p* < 0.05). The improved hepatic health in low-animals was supported by low HC and HT levels (*p* < 0.05). In CvitE animals, low inflammation correlated with reduced steatosis, ballooning cells and NAS-index (*p* < 0.01), as well as reduced hepatic HC and HT (*p* < 0.05). Fibrosis was not decreased in low compared to intermediate or high groups. Figure 4 displays correlation analyses and Figure 5 shows representative histological images. A correlation between inflammation score and CYP7A1 expression and fibrotic target gene expression (α-sma, Col1a1 and TGF-β) was detected in the Chow group (*p* < 0.05), although no differences in the expression level between sub-groups were observed. For the remaining investigated genes, there was no significant correlation between expression patterns and inflammation status of the subgroups (data not shown).

## 4. Discussion

Changing from a high-fat/cholesterol to a low-fat/no cholesterol diet improved dyslipidemia and hepatic health (hepatic lipid accumulation, liver weight and liver injury marker AST). High dose vitE supplementation resulted in a significantly reduced hepatocellular ballooning score compared with HF, although no significant effect was detected on the remaining hepatic hallmarks of NASH or in the expression of key target genes. Importantly, subgroup analyses showed an association between hepatic inflammation and several disease markers for both interventions, emphasizing inflammation as a key factor in NASH resolution and identifying distinct differences in sensitivity between subjects towards intervention.

In the Pioglitazone versus Vitamin E versus Placebo for the Treatment of Nondiabetic Patients with Nonalcoholic Steatohepatitis (PIVENS) trial, 800 IU/day vitE was administered to nondiabetic, adult NASH patients for 96 weeks. The study showed that vitE combined with life style modification recommendations (dietary modification, weight loss and exercise) has the ability to improve overall NASH, lowering liver injury enzymes (ALT and AST), and significantly improving hepatic steatosis, lobular inflammation and ballooning hepatocytes, although with no significant effect on fibrosis [20,26,45]. In the present study, the initial analyses did not show significant improvements in hepatic histopathology and additional markers in the intervention groups. This may well be due to the more advanced preintervention disease phenotype and the short intervention time (8 weeks) compared with the PIVENS trial. However, CvitE did show a small but positive and significant effect on ballooning hepatocytes, compared with animals from the preintervention (week 25) HF group. As a progressive disease, NASH severity is expected to advance from week 25 to 33, rendering the recorded difference likely to be underestimated compared to the expected difference had week 33 HF counterparts been included. The reduction in ballooning hepatocytes following vitE supplementation was also observed in a previous study in NASH guinea pigs exposed to eight weeks of vitE and atorvastatin therapy combined with diet intervention [20]. In addition, the beneficial effects on dyslipidemia, hepatic lipid accumulation, steatosis, NAS, and down regulation of inflammatory (IL-8, MCP-1, TNF-α) and fibrogenic (TGF-β, Col1a1, α-sma) target genes were recorded, supporting a positive outcome of the combination of diet change and vitE supplementation in NASH management [20]. In the present study, mRNA expression of fibrogenic markers was not significantly different between Chow, CvitE and HF groups. However, protein levels and cellular localization were not measured, thus putative functional consequences, e.g., on the number of activated (α-sma positive) hepatic stellate cells could not be assessed. VitE administration has been shown in mice to increase adiponectin levels, in turn increasing peroxisome proliferator-activated receptor α expression and the mitochondrial oxidative capacity, linking the antioxidant effects of vitE to decreasing oxidative stress and adhering antilipotoxic, anti-inflammatory and antifibrogenic properties [46,47].

Subanalyses of Chow and CvitE revealed a distinct association between the stage of hepatic inflammation and both histopathological, biochemical and molecular disease markers. Categorizing by inflammatory status identified that animals with a low degree of hepatic inflammation responded significantly better to the intervention in most of the analyzed outcomes. It can be speculated that the current intervention was sufficient to improve hepatocellular function including replenishing antioxidant capacity through vitE in milder cases of NASH, whereas more advanced disease stages require increased measures, e.g., expanded intervention times. However, the current finding of significant improvements supports a pivotal role of hepatic inflammation as a key factor in the resolution of NASH, pinpointing inflammation as a relevant therapeutic target and potential biomarker of NASH [48]. The differences in inflammatory status between animals within both intervention groups supports a difference in individual responsiveness, hence responders versus nonresponders [49,50]. Nonresponders/responders were also identified from the PIVENS trial, with additional analyses suggesting circulating metabolites as biomarkers for predicting patient sensitivity and NASH treatment outcome [26,51]. In this exploratory pilot study of a subset of samples from the PIVENS trial, vitE responders displayed decreased levels of the metabolite sphingosine, which is hypothesized to reflect and correlate with decreased TNF-α activity, hereby supporting inflammation as a target in NASH resolution and a potential biomarker of disease severity [51]. The current interventions improved histopathological features and expression of target genes that correlated with reduced HC and HT in subgroups of both Chow and CvitE groups, indicating increased excretion and clearance of cholesterol and triglycerides in these animals. This is supported by the correlation with increased CYP7A1 expression in Chow subgroups, which indicates that the conversion of cholesterol to bile acids is increased [37]. Min et al. showed CYP7A1 to be downregulated in liver samples of NASH patients compared to healthy controls and reported alterations in cholesterol and bile acid homeostatic pathways to be linked to disease severity [52]. A dysregulated cholesterol and bile acid metabolism in NASH patients is believed to increase the risk of hepatic injury and to correlate with NASH progression [53,54]. In the current study, ALP levels were not different between groups at euthanasia indicating that bile transport was not compromised. The measurement of circulating and hepatic bile acids could have provided further insight to bile metabolism and the potential functional consequences of the recorded CYP7A1 expression, however, it was not included in the present study.

Spontaneous regression of NASH and fibrosis has been reported in patients and occurs at unknown rates [3], and could be another explanation for the subgroup of animals showing overall improvement in the NASH profile. Although it is debated whether spontaneous regression as reported in patients is a direct reflection of the dynamic nature of NASH or if potential sampling error and inter- and intraobserver variability in sequential liver biopsies may limit the validity of the recorded findings [55,56].

The supplementation with high dose vitE lead to relatively subtle improvements compared to the chow diet. This supports that the change from a high-fat diet to a low-fat no cholesterol is a primary driver of the observed improvements and that increasing the vitE dose only has a modest effect on disease associated end points. In the management of NASH, life style modifications are mandatory and have previously been shown to increase the effect of pharmacotherapy [20,24]. Studies focusing on life style changes have shown beneficial effects on NAFLD, however studies investigating the effect on progressive NASH with advanced fibrosis are limited [57]. By introducing calorie restriction and physical activity, hepatic lipid accumulation and steatosis are rapidly affected by a weight loss of 3–5% [58], but to gain a significant effect on hepatic hallmarks of NASH and fibrosis a weight loss above 10% is necessary [21,59]. Positive effects on fibrosis were reported for patients with baseline scores less than 2, therefore not representative of advanced fibrosis, defined as a score of 3 or above [60]. In accordance, the introduction of a diet intervention in the present study resulted in a reduction in dyslipidemia, hepatic lipid accumulation and liver weight. A recent systematic literature review found that low-calorie diets improve the inflammatory profile in patients diagnosed with NAFLD [61]. An overall decrease in C-reactive protein, TNF-α and IL-6 and an increase in adiponectin levels, indicated an improvement in the inflammatory and possibly the oxidative status of NAFLD patients [61]. The authors suggest chronic inflammatory response in NAFLD/NASH as a putative therapeutic target area, and also propose inflammatory cytokines and adipokines as noninvasive markers of disease [61]. This is supported by previous studies showing a correlation between disease activity and serum TNF-α and adiponectin levels in children and adult biopsy-proven NAFLD patients warranting inflammation and oxidative stress as viable therapeutic targets of NASH [62,63,64]. Inhibition of TNF-α expression in a murine model resulted in significantly reduced hepatic steatosis and fibrosis, together with decreased expression of MCP-1, TGF-β, Col1a1 and metallopeptidase inhibitor 1 [65]. Conversely, the presence of TNF-α induced the formation of hepatic lesions and expression of the aforementioned target genes, supports TNF-α and subsequent inflammatory signaling to play a pivotal role in the development and progression of NASH [65].

## 5. Conclusions

Dietary intervention with and without high-dose vitE treatment resulted in an improved dyslipidemic profile and significantly reduced hepatic lipid accumulation and liver weight. Moreover, high-dose vitE administration resulted in an improvement in hepatocellular ballooning. Subanalyses revealed individual responses within both intervention groups according to inflammatory status; a low inflammatory response correlating with improved hepatic histopathological profile and decreased biochemical and molecular disease markers. Together, these results indicate that the therapeutic response to diet intervention and vitE supplementation may depend on hepatic inflammatory status and suggest inflammation and antioxidant status as relevant targets for the development of future therapeutic strategies in NASH.

## Figures and Tables

**Table 1:** Dietary composition.

Nutrient	HF	Control	Chow	CvitE
Protein (%)	16.7	16.8	16.8	16.8
Carbohydrates (%)	37.9	47.1	47.1	47.1
Fat (%)	20	4	4	4
Cholesterol (%)	0.35	-	-	-
Sucrose (%)	15	-	-	-
Vitamin E (all-rac-alpha-tocopherylacetate (mg/kg feed))	125	125	125	1125

HF, high-fat diet; Control, low-fat diet, also including Control25; Chow, low-fat diet; CvitE, low-fat diet supplemented with 1125 mg/kg feed vitamin E.

**Table 2:** *q*PCR primer details.

Gene	Accession No.	Forward (5′-3′)	Reverse (3′-5′)	Product (bp)
*IL-8*	NM_001173399.2	GGCAGCCTTCCTGCTCTCT	CAGCTCCGAGACCAACTTTGT	67
*TNF-α*	NM_001173025.1	GCCGTCTCCTACCCGGAAAA	TAGATCTGCCCGGAATCGGC	203
*MCP-1*	NM_001172926.1	TGCCAAACTGGACCAGAGAA	CGAATGTTCAAAGGCTTTGAAGT	75
*CYP7A1*	GQ507494.1	CTGGAGAAGGCAGGTCAACA	CTCCTTAGCTGTCCGGATGT	150
*TGF-β*	NM_001173023.1	AACCCGAGCCGGACTACTATG	TGCTTTTATAGATATTGTGGCTGT TGT	78
*col1a1*	XM_003466865.2	CTGGACAGCGTGGTGTAGTC	TCCAGAAGGACCTTGTTTGC	104
*α-sma*	ENSCPOT00000011693.2	GACATCAAGGAGAAGCTGTG	GCTGTTGTAGGTGGTTTCAT	273

IL-8, interleukin 8; TNF-α, tumor necrosis factor α; MCP-1, monocyte chemotactic protein 1; CYP7A1, cytochrome P450 7A1; TGF-β, transforming growth factor β; col1a1, collagen 1a1; α-sma, α smooth muscle actin.

**Table 3:** Plasma values.

	HF	Control 25	Control	Chow	CvitE
TC (mmol/L) ^j^	3.77 (2.65–5.35)	0.82 (0.46–1.48) ^####^	0.70 (0.53–0.94) ^####^	2.08 (1.69–2.55) ^#,^****	2.48 (1.95–3.16) ****
TG (mmol/L) ^j^	0.53 (0.44–0.65)	2.73 (1.04–7.19) ^####^	1.43 (1.16–1.78) ^####^	0.44 (0.40–0.48) ****	0.47 (0.43–0.51) ****
FFA (mmol/L)	0.51 ± 0.16	0.79 ± 0.19 ^#^	0.68 ± 0.19	0.58 ± 0.15	0.59 ± 0.19
ALP (U/L)	28.42 ± 4.66	39.67 ± 1.97 ^#^	34.00 ± 10.33	34.24 ± 6.47	33.67 ± 7.27
ALT (U/L)	63.11 ± 17.77	40.67 ± 15.32	32.67 ± 8.74 ^###^	52.19 ± 18.84 *	52.38 ± 22.99 *
AST (U/L)	546.0 ± 263.1	139.2 ± 231.3 ^###^	87.48 ± 57.98 ^####^	273.1 ± 133.5 ^##^	322.2 ± 198.6 ^#,^**
αToc (µmol/L) ^j^	2.17 (1.52–3.08)	1.58 (0.74–3.41)	1.19 (0.77–1.85)	1.98 (1.56–2.50)	6.89 (5.25–9.06) ^####,^****^,††††^

TC, total cholesterol; TG, triglyceride; FFA, free fatty acids; ALP, alkaline phosphatase; ALT, alanine aminotransferase; AST, aspartate aminotransferase; αToc, alpha-tocopherol/vitamin E. Data are presented as mean ± standard deviation. ^j^ Data are presented as geometric mean with 95% confidence intervals. * *p* < 0.05, ** *p* < 0.01, **** *p* < 0.0001, compared with Control. ^#^ *p* < 0.05, ^##^ *p* < 0.01, ^###^ *p* < 0.001, ^####^ *p* < 0.0001, compared with HF. ^††††^ *p* < 0.0001, compared with Chow.

**Table 4:** Liver values.

	HF	Control25	Control	Chow	CvitE
Liver weight (%) ^j^	6.63 (5.79–7.87)	2.62 (2.28–3.0) ^####^	2.65 (2.47–2.87) ^####^	4.37 (4.03–4.85) ^####,^****	4.57 (4.18–5.14) ^####,^****
HC (µmol/g)	80.09 ± 26.47	32.38 ± 21.34 ^####^	20.88 ± 17.77 ^####^	47.19 ± 15.74 ^###,^**	48.00 ± 17.65 ^###,^**
HT (µmol/g)	43.20 ± 9.64	5.48 ± 0.61 ^####^	5.06 ± 0.55 ^####^	23.75 ± 5.15 ^####,^****	24.73 ± 6.84 ^####,^****
αToc (nmol/g) ^j^	4.08 (3.08–5.4)	7.09 (3.45–14.57)	2.91 (2.11–4.01)	7.55 (5.52–10.32) **	75.22 (49.03–115.4) ^####,^****^,††††^

HC, hepatic cholesterol; HT, hepatic triglyceride; αToc, alpha-tocopherol/vitamin E. Data are presented as mean ± standard deviation. ^j^ Data are presented as geometric mean with 95% confidence intervals. ** *p* < 0.01, **** *p* < 0.0001, compared with Control. ^###^ *p* < 0.001, ^####^ *p* < 0.0001, compared with HF. ^††††^ *p* < 0.0001, compared with Chow.
